# Dietary flavonoid intake and psychological well-being – A bidirectional relationship

**DOI:** 10.1016/j.clnu.2026.106579

**Published:** 2026-01-19

**Authors:** Alysha S. Thompson, Nicola P. Bondonno, Yan Lydia Liu, Farah Qureshi, Laura D. Kubzansky, Claudia Trudel-Fitzgerald, Julia K. Boehm, Eric B. Rimm, Aedín Cassidy

**Affiliations:** a Co-Centre for Sustainable Food Systems and The Institute for Global Food Security, Queen's University Belfast, Northern Ireland, UK; b Danish Cancer Institute, Copenhagen, Denmark; c Nutrition & Health Innovation Research Institute, School of Medical and Health Sciences, Edith Cowan University, Joondalup, WA, Australia; d Department Nutrition, Harvard T.H Chan School of Public Health, Boston, MA, USA; e Department of Population, Family & Reproductive Health, Johns Hopkins Bloomberg School of Public Health, Baltimore, MD, USA; f Department of Social and Behavioral Sciences, Harvard T.H. Chan School of Public Health, Boston, MA, USA; g Lee Kum Sheung Center for Health and Happiness, Harvard T.H. Chan School of Public Health, Boston, MA, USA; h Department of Psychology, Université du Québec à Trois-Rivières, Trois-Rivières, Quebec, Canada; i Research Center, Institut Universitaire en Santé Mentale de Montréal, Montreal, Quebec, Canada; j Department of Psychology, Chapman University, Orange, CA, USA; k Channing Division of Network Medicine, Brigham and Women's Hospital and Harvard Medical School, Boston, MA, USA; l Department of Epidemiology, Harvard T.H. Chan School of Public Health, Boston, MA, USA

**Keywords:** Flavonoids, Flavodiet score, Flavonoid-rich foods, Psychological well-being, Happiness, Optimism

## Abstract

**Background and aim:**

Higher dietary flavonoid intake has been associated with lower risks of mortality and major chronic disease, yet its relationship with psychological well-being (PWB), a key contributor to health and quality of life, remains unclear. This study aimed to investigate bidirectional associations between dietary flavonoid intake and two PWB facets: happiness (positive emotional state) and optimism (generalized expectation of positive outcomes). Specifically, we examined whether (1) overall flavonoid-rich dietary patterns (flavodiet score), (2) intake of specific flavonoid-rich foods, and (3) total flavonoid and subclass intakes were each associated with happiness and optimism over time.

**Methods:**

Data were drawn from the Nurses’ Health Study to form two analytical samples. Flavonoid intake measured in 1990 (n = 44,659) was examined in relation to sustained happiness (1992–2000) while intake in 2002 (n = 36,723) was analysed in relation to sustained optimism (2004–2012). Secondary analyses assessed whether higher baseline levels of each PWB facet were associated with sustained higher flavonoid intake, over up to 18 years. Associations were assessed using generalized estimating equations, adjusting for potential confounders.

**Results:**

Higher flavodiet scores were associated with a 3–6 % higher likelihood of sustained happiness [RR_Q4vsQ1_ (95 % CI): 1.03 (1.02–1.05)] and optimism [RR_Q4vsQ1_ (95 % CI): 1.06 (1.01–1.11)]. Specific flavonoid-rich foods (strawberries, apples, oranges, grapefruit, blueberries) were associated with a 3–16 % greater likelihood of sustained PWB, across the two facets. Similarly, total flavonoid and subclass intakes were associated with a 2–18 % greater likelihood of sustained PWB. Women with higher baseline levels of happiness or optimism were also more likely to sustain a higher flavonoid intake.

**Conclusions:**

Consuming ~3 servings/day of flavonoid-rich foods is associated with sustained PWB, and higher baseline PWB is associated with sustained higher flavonoid intake over up to 18 years. This bidirectional relationship suggests that integrated interventions targeting both diet and well-being may help promote long-term health and reduce chronic disease risk.

## 1. Introduction

Psychological well-being (PWB), an overarching construct encompassing dimensions and expectancies such as happiness, optimism, and having a sense of purpose, is increasingly recognised as a central component of overall health and quality of life [1-3]. Beyond its intrinsic value, high PWB has been associated with numerous health benefits including lower risks of chronic disease and mortality, as well as greater healthspan [2,4-8]. Pathways by which high PWB may protect physical health, include associations with healthier behaviours [4,8-10]. Importantly, there is also evidence that the associations between PWB and health behaviours are bidirectional [11], whereby healthier behaviours can also lead to higher PWB.

Previous research has explored the relationship between dietary patterns and adverse mental health outcomes, such as depression, suggesting a beneficial role of consuming fruits, vegetables and whole grains [12-16]. These foods are typically nutrient-dense and rich in bioactive compounds, particularly flavonoids—a class of polyphenolic compounds found abundantly in fruits (such as berries, apples, and citrus), vegetables (e.g., onions, kale), tea, cocoa, and red wine. Flavonoids have been shown to protect against cognitive aging, dementia, and mental health disorders [17,18] through several mechanisms, including modulation of neuroinflammation, regulation of enzyme activity, and enhancement of cellular functions such as cerebrovascular blood flow and synaptic plasticity [19]. Additionally, following ingestion, flavonoids are metabolised by the gut microbiome into more bioactive, gut-derived metabolites [20]. These metabolites can influence the production of key molecules such as short-chain fatty acids (SCFA), which may play a role in modulating neuroinflammatory processes and neurotransmitter pathways [21,22]. Furthermore, in preclinical studies, flavonoids have been shown to promote increased expression of neurotransmitters, such as serotonin, dopamine, and gamma-aminobutyric acid, which are key regulators of mood, as well as neurotrophic factors like brain-derived neurotrophic factor, which support neurogenesis and brain plasticity [23,24]. While these processes are generally linked to better mood and cognitive function, it is plausible that flavonoids contribute to PWB by supporting brain health and resilience.

Although much of the existing research has focused on the predictive value of psychological factors, such as anxiety and depression symptoms, in relation to subsequent dietary patterns, there is a growing interest in studying positive health assets [25]. Although studies have begun to explore how dietary patterns relate to PWB [26], few have examined the potential role of bioactive dietary components, such a flavonoids, which are known to support brain health.

We therefore explored the associations between: (1) primary dietary sources of flavonoid intake, as reflected by a composite flavodiet score, (2) consumption of flavonoid-rich foods, and (3) intake of specific flavonoid subclasses and two dimensions of PWB, namely, happiness and optimism, over 10 years among women in the Nurses’ Health Study (NHS). Primary analyses examined baseline flavonoid intake in relation to sustained levels of happiness and optimism. Secondary analyses explored potential bidirectionality in the associations between flavonoid intake and PWB, given prior evidence suggesting that diet and PWB may influence each other over time. Additionally, given that body weight may influence both dietary patterns and PWB, stratified analyses were conducted by baseline BMI (<25 vs. ≥25) to assess potential effect modification.

## 2. Methods

### 2.1. Study population

The NHS is a long-term, U.S-based cohort study that began in 1976, enrolling 121,700 female registered nurses aged 30–55 [27]. Since inception, the NHS has measured participants’ lifestyle factors, medical history, and newly diagnosed health conditions via biennial mailed questionnaires, maintaining a follow-up rate exceeding 85 % [28].

This study examined associations between dietary flavonoid intake and two dimensions of PWB—happiness and optimism—measured at different times in the NHS, thus requiring two separate analytical samples. While dietary intake has been assessed every four years since the study's inception, measures of PWB facets were administered less frequently and not simultaneously across all cycles. Specifically, happiness and optimism were assessed on alternating four-year cycles via biennial questionnaires, which limited the possibility of using a uniform analytic baseline across analyses. For the primary happiness analyses, dietary intake reported in 1990 was used as the analytic baseline and examined in relation to repeated happiness assessments over three follow-up periods: 1992, 1996, and 2000. For the primary optimism analyses, dietary intake reported in 2002 was used as the analytic baseline and examined in relation to repeated optimism assessments across three follow-up periods: 2004, 2008 and 2012. For further details, see Supplementary Table S1.

**Fig. 1.**
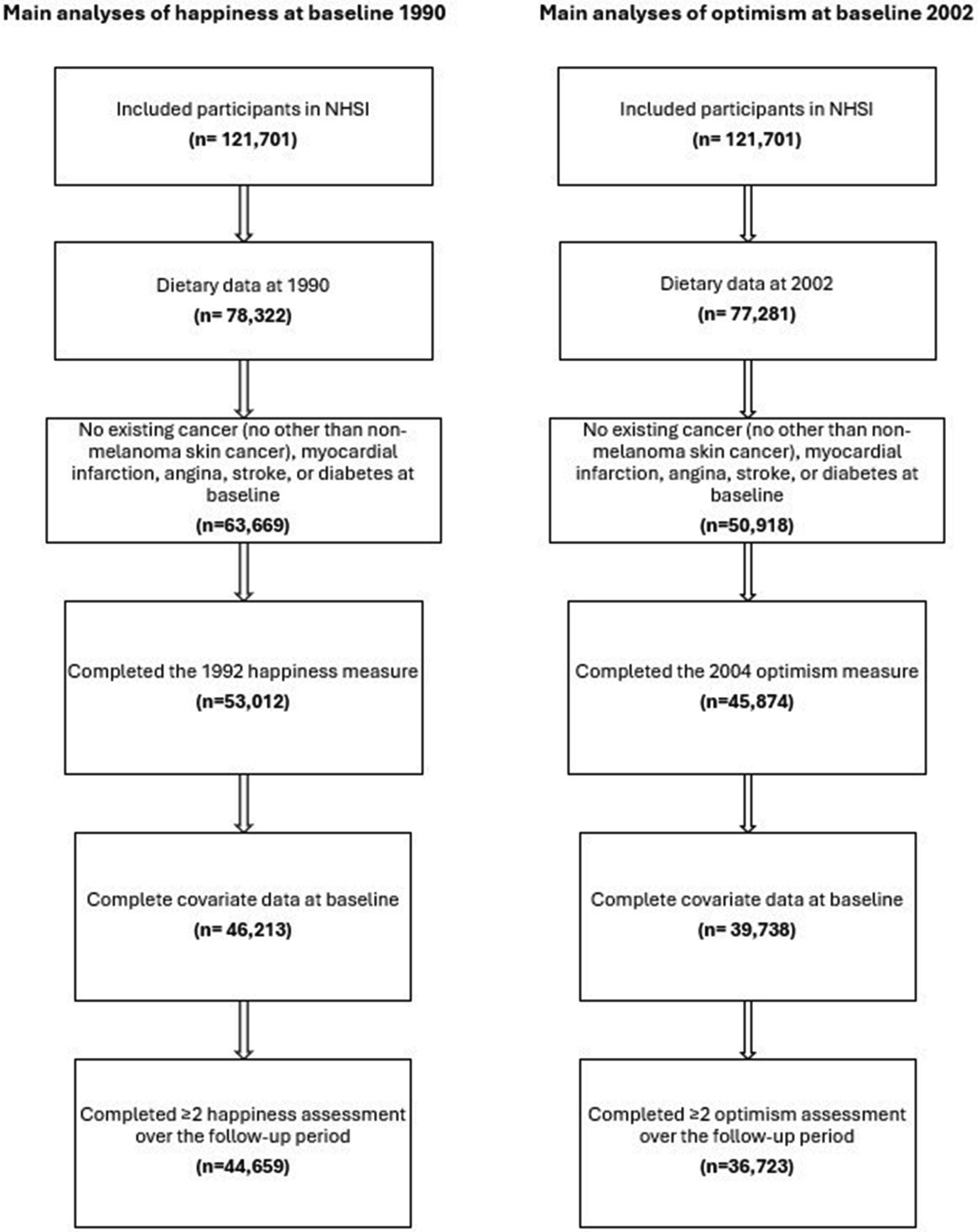
Flowchart of Nurses' Health Study participants included in final analyses.

Women were excluded from the analysis if they had missing dietary intake data at the respective analytical baseline (1990 for happiness analyses and 2002 for optimism analyses). Following prior work examining relationships between PWB and lifestyle behaviours [11,29], participants were also excluded if they reported a major chronic condition at baseline (such as cancer [excluding non-melanoma skin cancer], diabetes, myocardial infarction, or angina), as these individuals may experience different levels of PWB and follow distinct dietary patterns compared to generally healthy women. Women were further excluded if they had missing baseline PWB data, reported PWB data for only one follow up, or had missing data on key covariates at the respective analytic baseline. For flavonoid-related exposures, participants were excluded if total flavonoid intake was missing at baseline. We also derived a composite flavodiet score, calculated as the sum of daily servings of seven flavonoid-rich foods and beverages (tea, apples, oranges, grapefruits, blueberries, strawberries, and red wine; see Section 2.2 for further details). To minimise unnecessary data loss, a multistep approach was applied to item-level missingness. First, if a participant reported a non-zero total intake for a broader food category (e.g., total fruit or total alcohol) but a specific flavodiet component within that category was missing (e.g., strawberries, apples, red wine), the missing item was assigned a value of zero. Missing tea intake was also set to zero. This step ensured that participants who reported category-level intake but omitted individual items were not excluded. Second, the flavodiet score was considered missing only when all component foods and beverages were missing. Finally, when constructing the analytic dataset, participants with any remaining missing values for flavodiet components, total flavonoid intake, flavonoid subclasses, or other nutrient intakes were excluded, resulting in a complete-case analysis for all dietary variables. The final analytic samples comprised 44,659 women for the happiness analyses and 36,723 for the optimism analyses (Fig. 1).

In secondary analyses examining associations in the other direction—i.e., the relationship between baseline PWB and sustained moderate-to-high flavonoid intake—happiness measured in 1992 was used as the analytic baseline and assessed in relation to repeated measures of flavonoid intake over five follow-up assessments from 1994 to 2010. Similarly, optimism measured in 2004 was used as the analytic baseline and analysed with repeated flavonoid intake assessments over two follow-up periods from 2006 to 2010. Sustained moderate-to-high flavonoid intake was defined as reporting intake in the upper two quartiles (Q3-Q4) at two or more timepoints during the study period, including baseline. Women with only a single dietary assessment during follow-up were excluded, resulting in sample sizes of 43,564 for the happiness analyses and 30,823 for the optimism analyses.

### 2.2. Assessment of dietary flavonoid intake

Participants from both analytical samples completed validated semi-quantitative food frequency questionnaires (SFFQs) at baseline and every four years thereafter [30,31]. The SFFQs assessed average frequency and portion size of consumption of specific food and drink items over the previous year, with response options ranging from “never or less than once a month” to “six or more times per day”. The frequency of consumption of flavonoid-rich foods was recorded as servings per day, week, or month. Flavonoid intake was calculated by multiplying the reported consumption frequency of each food item by its flavonoid content, derived from the U.S Department of Agriculture (USDA) flavonoid and proanthocyanidin databases [32,33]. These data were used to construct a NHS-specific flavonoid database, from which time-updated intake estimates (mg/d) for total flavonoid intake and six flavonoid subclasses (flavonols, flavan-3-ol monomers, flavan-3-ol polymers (including proanthocyanidins, theaflavins, and thearubigins), anthocyanins, flavanones, and flavones) were derived, as previously described [34]. Major dietary sources of flavonoids (such as apples, tea, and red wine), along with total flavonoid intake and most flavonoid subclasses (excluding flavones), have demonstrated good reproducibility and validity within the NHS over time [35,36].

The exposures of interest included: (1) a composite flavodiet score, calculated by summing the daily servings of seven flavonoid-rich foods and beverages that each contributed more than 1 % to flavonoid intake–namely tea, apples, oranges, grape-fruits, blueberries, strawberries, and red wine–consistent with prior NHS-based research [37,38]. This score represents a food-based indicator of adherence to a flavonoid-rich dietary pattern; (2) intake of each individual food item included in the flavodiet score; and (3) total flavonoid intake and the six major flavonoid subclasses commonly consumed in the U.S diet (flavonols, flavan-3-ol monomers, flavan-3-ol polymers [including proanthocyanidins, theaflavins, and thearubigins], anthocyanins, flavanones, and flavones), expressed in mg/day, derived from the NHS flavonoid database and defined as the sum of subclass intakes.

### 2.3. Ascertainment of psychological well-being

In 1992, 1996, and 2000, participants were asked a single item question from the Medical Outcomes Study Questionnaire Short Form 36 Health Survey (SF-36) [39]: “How much of the time during the last month have you been a happy person?”. Response options, which ranged from 1 (“all the time”) to 6 (“none of the time”), were reverse-coded, with higher values indicating greater happiness. Although single-item measures of PWB are inherently limited, they have been shown to perform comparably to multi-item measures as well as to be predictive of behavioural and health outcomes [40,41]. In this study, the reproducibility of happiness scores over time was moderate, with an intra-class correlation coefficient (ICC) of 0.44.

In 2004, 2008, and 2012, optimism was assessed using the validated 6-item Life Orientation Test-Revised [42]. Participants rated their agreement with statements reflecting a positive outlook on life such as, “In uncertain times, I usually expect the best.” Negatively worded items were reverse-coded and all responses were summed to yield a total score ranging from 6 to 30, with higher scores indicating greater optimism. The ICC for optimism scores over time was 0.63, indicating good temporal stability. Cronbach's alpha values at each assessment also demonstrated good internal consistency (α_2004_ = 0.78; α_2008_ = 0.76; α_2012_ = 0.76).

Building on previously established methods [11,29,43,44], the primary analyses used two binary (yes/no) variables to identify women who sustained high levels of happiness and optimism, separately, across the study period. Sustained high happiness was defined as reporting high levels (i.e., “all” or “most” of the time) on at least two of three assessments, including baseline (1992, 1996, and 2000). Specifically, happiness responses were based on a 6-point scale and categorised into tertiles: “higher” (all or most of the time; responses 1–2), “moderate” (a good bit of the time; response 3), and “lower” (some, little, or none of the time; responses 4–6). Only those in the highest tertile at two or more time points were classified as having sustained high happiness. For optimism, the variable was derived from a 6-item measure, scored from 0 to 30, and divided into tertiles: lower (0–23), moderate (24–27), and higher (28–30). Sustained high optimism was defined as scoring in the highest tertile (28–30) at two or more time points (2004, 2008, and 2012), including the baseline assessment. In sensitivity analyses, a stricter definition of sustained happiness and optimism was applied, requiring high levels at all three respective time points. For secondary analyses, baseline happiness and optimism were categorised into three levels (lower, moderate, higher) based on the distribution of scores within each analytical sample to explore potential dose-response relationships across the well-being continuum and to ensure robustness of findings beyond binary classification. Consistent with previous cohorts [45], the distribution of happiness scores was uneven (1992 happiness levels in the flavodiet score analysis: lower = 14 %; moderate = 19 %; higher = 67 %). In contrast, optimism scores were more evenly distributed (2004 optimism levels for the flavodiet score analysis: lower = 31 %; moderate = 31 %; higher = 38 %). The correlation between baseline happiness and optimism was r = 0.25.

### 2.4. Ascertainment of covariates

Covariates included in this study were all self-reported, assessed via questionnaires at the analytic baseline and updated every two years thereafter. Participants reported their demographic characteristics, including baseline age (continuous, calendar years), ethnicity (non-Hispanic White; underrepresented minorities), education level (registered nurse; bachelor; master's; doctorate), and marital status (married/in a relationship; unmarried/not in a relationship). Health and lifestyle factors included smoking status (never; former; current), multivitamin use (no; yes), physical activity (continuous, metabolic equivalent-hours/week [46], body mass index (BMI), computed from self-reported height and weight (continuous, kg/m^2^) [47], menopausal status, based on self-report of natural cessation of menstrual periods, bilateral oophorectomy, or hysterectomy (premenopausal; postmenopausal) [48], postmenopausal hormone use (never; past; current), and depression defined as regular antidepressant use in the past two years or physician-diagnosed depression. Dietary covariates were assessed using the SFFQ and updated every four years. These included alcohol intake, total energy intake, and intakes of meat, nuts, saturated fat, polyunsaturated fat, trans fat, cereal fibre, and soft drinks, consistent with previous NHS studies on flavonoids and health outcomes [37,38].

### 2.5. Statistical analysis

In primary analyses, multivariable-adjusted generalized estimating equations (GEE) with a modified Poisson distribution and robust (sandwich) variance estimators—used due to an outcome prevalence >10 % [49]—were employed to assess associations of baseline flavonoid intake measured in three different ways, through the flavodiet score, flavonoid-rich foods, and flavonoid subclasses, with sustained levels of happiness or optimism over the study period. To account for multiple testing, P-values were adjusted using the Benjamin-Hochberg method (P-corrected). Two models were used across all analyses: Model 1 adjusted for age, race/ethnicity, education and marital status, while Model 2 further adjusted for smoking status, multivitamin use, physical activity, BMI, menopausal status, postmenopausal hormone use, and intakes of alcohol, total energy, meat, nuts, saturated fat, polyunsaturated fat, trans fat, cereal fibre, and soft drinks.

Secondary analyses employed similarly structured, increasingly adjusted GEE models to explore potential bidirectionality in the associations. Specifically, they examined baseline PWB, happiness (measured in 1992) and optimism (measured in 2004), in relation to sustained moderate-to-high flavonoid intake over time. Sustained intake was defined as remaining in the top two quartiles (Q3-Q4) for both the flavodiet score and total flavonoid intake across two or more assessments, including baseline: from 1994 to 2010 for the happiness analyses, and from 2006 to 2010 for the optimism analyses. Baseline PWB was categorized as lower, moderate, or higher.

To further explore heterogeneity, we conducted stratified analyses to test for potential effect modification by baseline BMI (<25 kg/m^2^ vs. ≥25 kg/m^2^). Effect modification was formally tested using interaction terms, with results also presented separately within strata of BMI. Two sensitivity analyses were then conducted to assess the robustness of the findings: (1) excluding participants with depression at baseline; and (2) applying a stricter definition of sustained happiness and optimism, which required higher levels at all three respective assessment timepoints.

## 3. Results

### 3.1. Baseline characteristics

At the 1990 baseline for happiness, the cohort included 44,659 women with a mean (range) age of 56.0 (43.0–70.0) years, while at the 2002 baseline for optimism, the cohort consisted of 36,723 women who were, on average, 66.6 (55.0–82.0) years (Table 1). Compared to women in the happiness analytic sample, those in the optimism analytic sample were more likely to be older, postmenopausal, use hormone therapy, take multivitamins, follow a healthier diet, and engage in more physical activity and not smoke. However, a higher proportion in the optimism analytic sample had a history of depression. In both samples, participants with a higher flavodiet score were more likely to be former or never smokers and to have higher energy intakes compared to those with lower flavodiet scores (Table 1).

### 3.2. Flavonoid intake and sustained high psychological well-being

After adjusting for sociodemographic, lifestyle and menopausal-related factors, compared to the lowest scores (Q1 median = 0.3 servings/day), a higher flavodiet score (Q4 median = 3.0 servings/day) was associated with a 3 % higher likelihood of sustained happiness [RR_Q4vsQ1_ (95 % CI): 1.03 (1.02–1.05), Table 2]. For optimism, a higher flavodiet score (Q4 median = 3.1 servings/day) was associated with a 6 % higher likelihood of sustained optimism [RR_Q4vsQ1_ (95 % CI): 1.06 (1.01–1.11)], Table 2], when compared to the lowest scores (Q1 median = 0.3 servings/day).

Of the individual flavonoid-rich foods, higher intakes of strawberries, apples, oranges, and grapefruit were associated with an 8 %, 3 %, 6 % and 6 % higher likelihood of sustained happiness over time, respectively, in fully adjusted models [RR_Q4vsQ1_ (95 % CI); strawberries: 1.08 (1.05–1.10); apples: 1.03 (1.01–1.05); oranges: 1.06 (1.04–1.08); grapefruit: 1.06 (1.04–1.08), Table 2]. Similarly, but with greater magnitude and evidence of a dose-response relationship, higher intakes of strawberries, blueberries, apples, oranges, and grape-fruit were associated with a 16 %, 14 %, 15 %, 10 % and 10 % higher likelihood of sustained optimism over time, respectively, after adjusting for all covariates [RR_Q4vsQ1_ (95 % CI); strawberries: 1.16 (1.10–1.22); blueberries: 1.14 (1.08–1.21); apples: 1.15 (1.09–1.21); oranges: 1.10 (1.05–1.46); grapefruit: 1.10 (1.05–1.15), Table 2].

After adjusting for demographic, lifestyle and menopausal-related factors, higher intakes of total flavonoids and flavonoid subclasses were associated with a greater likelihood of sustained happiness over time. Specifically, comparing the highest quartile (Q4) to the lowest quartile (Q1), total flavonoids were associated with a 3 % higher likelihood of sustained happiness [RR_Q4vsQ1_ (95 % CI): 1.03 (1.01–1.05)]. Additionally, higher intakes of the flavone, flavanone, anthocyanin, and flavonol subclasses were associated with a 10 %, 9 %, 6 %, and 2 % higher likelihood of sustained happiness over time, respectively [RR_Q4vsQ1_ (95 % CI); flavones: 1.10 (1.08–1.12); flavanones: 1.09 (1.07–1.11); anthocyanins: 1.06 (1.04–1.08); flavonols: 1.02 (1.00–1.04), Table 3]. Furthermore, higher intakes of total flavonoids were associated with a 9 % higher likelihood of sustained optimism [RR_Q4vsQ1_ (95 % CI): 1.09 (1.04–1.15)], while the flavonol, flavone, flavanone, flavan-3-ol monomer, and anthocyanin subclasses were each associated with a 6–18 % higher likelihood of sustained optimism [RR_Q4vsQ1_ (95 % CI); flavonols: 1.15 (1.09–1.21); flavones: 1.18 (1.12–1.24); flavanones: 1.13 (1.08–1.18); flavan-3-ol monomers: 1.06 (1.01–1.11); anthocyanins: 1.16 (1.11–1.23), Table 3].

### 3.3. Psychological well-being and sustained flavonoid intake

Compared to women with lower levels of happiness, women with moderate or higher levels were more likely to sustain higher flavonoid intakes over 18 years of follow-up [flavodiet score RR_moderate_: 1.04 (1.01–1.07); RR_higher_: 1.06 (1.03–1.08); total flavonoid intake RR_moderate_: 1.04 (1.01–1.07); RR_higher_: 1.05 (1.03–1.07)], after multivariable adjustments (Table 4). Similarly, but with a slightly greater magnitude, women with moderate or higher (versus lower) levels of optimism were also more likely to sustain higher flavonoid intakes over a 6-year follow-up [flavodiet score RR_moderate_: 1.09 (1.05–1.13); RR_higher_: 1.09 (1.05–1.13); total flavonoid intake RR_moderate_: 1.06 (1.02–1.10); RR_higher_: 1.05 (1.01–1.08), Table 4].

### 3.4. Stratified analyses

Associations between the flavodiet and total flavonoid intake and sustained happiness or optimism did not significantly differ across strata of BMI (<25 kg/m^2^ vs. ≥25 kg/m^2^) (all P-interaction >0.1). However, associations with optimism appeared somewhat stronger among participants with BMI ≥25. For example, higher flavodiet scores were associated with a greater likelihood of sustained optimism among women with BMI ≥25 kg/m^2^ [RR_Q4vsQ1_ (95 % CI): 1.07 (1.00–1.14)], compared with weaker associations among those with BMI <25 kg/m^2^ [RR_Q4vsQ1_ (95 % CI): 1.04 (0.97–1.12)]. A similar pattern was observed for total flavonoid intake [BMI ≥25 kg/m^2^: 1.14 (1.06–1.22); BMI <25 kg/m^2^: 1.03 (0.96–1.11)] (Supplementary Table S2).

### 3.5. Sensitivity analyses

Excluding participants with depression at baseline did not materially change the associations between the flavodiet score, total flavonoid intakes, and either happiness [RR_Q4vsQ1_ (95 % CI); flavodiet score: 1.03 (1.01–1.05); RR_Q4vsQ1_ (95 % CI); total flavonoids: 1.02 (1.00–1.04)], or optimism [RR_Q4vsQ1_ (95 % CI); flavodiet score: 1.05 (1.00–1.11); RR_Q4vsQ1_ (95 % CI); total flavonoids: 1.08 (1.03–1.14), Supplementary Table S3].

After applying a stricter definition of sustained happiness and optimism, requiring high levels at all three respective assessment timepoints, associations between the flavodiet score and sustained happiness were marginally stronger [RR_Q4vsQ1_ (95 % CI): 1.06 (1.03–1.09)], as were associations with total flavonoid intake [RR_Q4vsQ1_ (95 % CI): 1.05 (1.02–1.08)]. In contrast, associations with sustained optimism showed no meaningful change: point estimates were similar to those in the main analysis, although confidence intervals widened and estimates were no longer statistically significant after full multivariable adjustment (Supplementary Table S4). Notably, moderate intakes of both exposures remained positively associated with sustained optimism, suggesting a potential threshold or non-linear relationship at higher intake levels.

## 4. Discussion

Our data suggest that a higher flavodiet score, reflecting a diverse intake of flavonoid-rich foods and beverages, is associated with a higher likelihood of sustaining high levels of happiness and optimism over a 10-year follow-up period. The associations were primarily driven by flavonoid-rich fruits such as strawberries, apples, oranges, grapefruit and blueberries, which were associated with both sustained high levels of happiness and/or optimism. Additionally, higher intakes of total flavonoids and their subclasses were also associated with a higher likelihood of sustained happiness and optimism over time. These results underscore the potential role of a flavonoid-rich diet in promoting some dimensions of PWB. While many of the associations observed were statistically significant, it is important to acknowledge that the effect sizes were modest. Nevertheless, even small improvements in PWB, when sustained at the population level, may translate into meaningful public health benefits. Notably, findings were generally consistent across both happiness and optimism although associations appeared slightly stronger with the more reliably assessed measure of optimism, suggesting robustness of the results across different PWB constructs. Moreover, bidirectional analyses suggest that PWB itself may influence subsequent flavonoid intake, as women with moderate or higher levels of happiness and optimism, respectively, were more likely to sustain greater flavonoid intakes over up to 18 years. Conversely, psychological distress may undermine flavonoid-rich dietary patterns by reducing motivation, increasing preference for energy-dense comfort foods, and impairing dietary self-regulation, all of which could lead to lower consumption of fruits and other flavonoid-rich foods. Such bidirectional relationships could contribute to positive feedback loops, potentially creating upward spirals of health and well-being over time.

Interestingly, we did not observe significant associations for flavonoid-rich drinks (tea and red wine) or for flavan-3-ol polymers, despite these sources contributing substantially to total flavonoid intake. Several factors may explain these null findings. Firstly, tea consumption in the NHS cohort is high but relatively homogeneous, resulting in limited between-person variability and reduced statistical power to detect associations. Secondly, polyphenol content in tea and red wine varies considerably by brewing method, type, storage, and serving size, which is not captured by the FFQs, potentially introducing non-differential measurement error and biasing associations toward the null [51]. Finally, consumption of these beverages may be more strongly shaped by cultural or habitual factors, making them less sensitive to variations in PWB than foods such as berries or citrus, which showed clearer associations.

The relationship between dietary flavonoid intake and PWB remains largely unexplored. However, both observational and interventional studies have examined the broader impact of diet on mental health outcomes [52,53]. For instance, Firth et al. [54] suggest that poor nutrition may contribute to low mood, whereas a diet rich in fibre, polyphenols, and unsaturated fatty acids may support both mental health (e.g., mood, stress and cognitive function) and physical health (e.g., insulin sensitivity, body weight, and other health-related behaviours). Similarly, Boehm et al. [55] report positive associations between optimism and serum antioxidant concentrations. These benefits are thought to stem at least in part from dietary influences on various biological processes related to health, including metabolic regulation, oxidative stress/anti-inflammatory activity [56-58], as well as gut microbiome modulation [59]. Further evidence supporting the potential role of diet on mental health comes from a previous U.S study, which suggests that flavonoid intake may help alleviate symptoms of depression and anxiety [60]. While direct evidence linking flavonoid intake to sustained PWB specifically is limited, these findings, along with broader research on diet and mental health, suggest a possible beneficial association. Moreover, they provide novel insight, as PWB is now recognized as more than the absence of psychological distress [1,61]. Accordingly, results from our sensitivity analyses excluding women with depression were overall robust to those obtained in our primary analyses, suggesting PWB's association with flavonoid intake is independent of psychological distress symptoms.

Our finding that higher PWB was associated with a greater likelihood of sustaining a flavonoid-rich dietary pattern aligns with theories of positive psychology. Positive psychological dimensions, such as happiness and optimism, are associated with enhanced motivation, self-regulation, and goal-directed behaviours, which in turn can foster greater adherence to health-promoting dietary habits [2]. For instance, individuals with higher optimism may perceive health-promoting behaviours as more achievable and rewarding, which reinforces long-term adherence to nutrient-rich diets [62]. Additionally, better emotional regulation among those with higher PWB may influence appetite and reduce emotional eating, further promoting healthier food preferences [63,64]. Socioeconomic and environmental factors likely act as important confounders in the observed associations. Higher PWB is often linked to greater access to resources, higher levels of education, and a supportive social environment [25,45] – all of which can facilitate healthier dietary habits. Conversely, individuals with limited socioeconomic resources may face financial and environmental barriers to consuming a diverse, flavonoid-rich diet. For example, the expense associated with fruits, vegetables, and other flavonoid-rich foods can limit access, particularly in areas with limited availability of fresh produce [11,65]. Despite this, in a RCT, more optimistic women showered greater improvements in diet quality, suggesting that optimism may promote healthier eating independently of socioeconomic status [43]. Improving PWB may represent a promising strategy to encourage greater consumption of flavonoid-rich foods, which are well-documented for their protective effects against a variety of chronic diseases.

The flavodiet score captures the consumption of multiple flavonoid-rich food sources, reflecting dietary patterns that incorporate distinct flavonoid subclasses. This diversity is important, as different flavonoid compounds exert unique biological effects, such as modulating inflammation, lowering oxidative stress, and enhancing endothelial function – all of which have previously been associated with improved brain health and mood regulation [19,66]. Furthermore, greater diversity of flavonoid sources may improve the bioavailability and synergistic activity of these compounds, optimising their overall health benefits [50,51]. These findings are consistent with recent evidence from the UK, which reported that greater flavonoid diversity, independent of total intake, was associated with a lower risk of mortality and major chronic diseases [67]. A diet abundant in diverse flavonoid-rich foods is typically nutrient-dense, offering a variety of vitamins, minerals, and bioactive compounds that collectively promote physical and psychological health [68]. Unlike total flavonoid intake, which may disproportionally reflect consumption of a limited range of foods (e.g., tea or berries), the flavodiet score offers clearer public health translation by encouraging a broader dietary approach. It highlights the importance of incorporating a wide variety of flavonoid-rich foods and beverages, which can help maximise nutrient intake and bioactive benefits while simultaneously providing a more sustainable and actionable dietary guideline for improving overall health outcomes.

Although statistical tests did not indicate significant effect modification by BMI, some differences in the associations between flavodiet or total flavonoid intake and sustained optimism were observed between BMI groups. Women with higher BMI appeared to have slightly stronger associations, which could reflect differences in baseline metabolic or inflammatory status. Higher BMI is often linked with greater systemic inflammation and oxidative stress [69], factors that have been associated with psychological symptoms [70,71]. Given this context, flavonoid-rich diets—characterised by anti-inflammatory and antioxidant properties—might show relatively stronger associations with positive psychological outcomes among individuals with higher BMI. These findings are exploratory and require confirmation, but they suggest that baseline metabolic or inflammatory status could influence the relationship between diet and PWB.

A notable strength of this study is its large, well-characterised cohort with long-term follow-up. Repeated measures of both diet and PWB further enabled robust analysis between dietary patterns, happiness, and optimism, and the ability to test for bidirectionality over time. However, several limitations must also be acknowledged. The NHS cohort primarily consists of non-Hispanic White, highly educated female health professionals. While this may enhance internal validity due to greater health awareness and access to flavonoid-rich foods, it limits the generalisability of findings to populations with lower educational attainment or more diverse socioeconomic and racial/ethnic backgrounds. Socioeconomic status may independently influence both dietary habits and PWB, potentially introducing residual confounding despite adjustment for multiple covariates in our analyses. Further, the use of a single-item question to assess happiness may reduce precision and may not fully capture the multidimensional nature of PWB. Nonetheless, previous studies have shown that happiness and optimism scores remain stable and reproducible over time [11,29]. Additionally, neither the flavodiet score nor total flavonoid intakes account for all potential sources of flavonoids, such as dark chocolate, herbs, spices, or other foods consumed in very small quantities, which were not consistently captured across FFQ cycles. They also do not consider variations in flavonoid content due to storage, processing, and preparation methods. Although major flavonoid-rich foods have demonstrated good validity and reproducibility within the NHS FFQs [35,36], any underestimation of flavonoid intake from concentrated sources consumed in small amounts, such as herbs and spices, would likely introduce non-differential misclassification and bias associations toward the null. Lastly, while our findings support a bidirectional association between flavonoid intake and PWB, distinguishing between the two directions of this relationship can only be definitively achieved in a clinical trial setting [43]. Future research should aim to replicate these findings in more diverse populations, including younger individuals and those from different racial, ethnic, and socioeconomic backgrounds. Furthermore, investigating the impact of socioeconomic barriers of flavonoid intake and PWB could provide valuable evidence to inform targeted public health initiatives.

## 5. Conclusion

Our findings highlight the potential of a flavonoid-rich diet, specifically consuming 3 servings/day of a variety of flavonoid-rich foods, to support long-term PWB. They also underscore the complex, bidirectional relationship between PWB and dietary behaviours, offering valuable insights for the development of integrated dietary and mental health strategies. Given the bidirectional nature of associations, simultaneously promoting PWB and the consumption of diverse flavonoid-rich foods may provide a comprehensive approach to improving overall health and reducing the burden of chronic diseases. Encouraging dietary diversity, particularly through the inclusion of a wide variety of flavonoid-rich fruits, may serve as a practical strategy to achieve these dual benefits.

## Supplementary Material

1

## Figures and Tables

**Table 1:** Age-standardised characteristics of women across quartiles (Q) of flavodiet score at baseline happiness and optimism levels.

	1990 Happiness Baseline (n = 44,659)			2002 Optimism Baseline (n = 36,723)						
	Q1 (n = 10,681)	Q2 (n = 11,341)	Q3 (n = 11,333)	Q4 (n = 11,304)	Whole sample (n = 44,659)	Q1 (n = 8448)	Q2 (n = 9212)	Q3 (n = 9501)	Q4 (n = 9562)	Whole sample (n = 36,723)
**Flavodiet score, serves/d**	0.4 (0.3)	0.9 (0.4)	1.5 (0.5)	3.2 (1.3)	1.5 (1.3)	0.6 (0.8)	1.0 (0.8)	1.6 (0.9)	3.0 (1.5)	1.6 (1.4)
**Age (years)** ^a^	55.3 (43.0–70.0)	56.1 (43.0–70.0)	56.4 (43.0–70.0)	56.0 (43.0–70.0)	56.0 (43.0–70.0)	66.8 (55.0–82.0)	66.7 (55.0–82.0)	66.6 (55.0–82.0)	66.3 (55.0–82.0)	66.6 (55.0–82.0)
**Race (%)**										
White	98.2	98.3	98.0	98.6	98.3	98.3	98.2	98.0	98.1	98.2
**Highest education level is RN degree (%)**	71.9	68.0	66.9	67.7	68.6	71.5	67.8	66.5	64.6	67.5
**Marital status (%)**										
Divorced, separated or widowed	18.6	16.5	15.9	15.6	16.6	24.6	22.1	22.7	22.0	22.8
Married or in a relationship	81.4	83.5	84.1	84.4	83.4	75.4	77.9	77.3	78.0	77.2
**BMI, kg/m^2^**	25.5 (4.8)	25.3 (4.5)	25.3 (4.5)	25.3 (4.6)	25.3 (4.6)	26.7 (5.2)	26.6 (5.0)	26.2 (4.7)	25.9 (4.7)	26.3 (4.9)
**Premenopausal (%)**	22.6	22.4	22.6	22.7	22.6	0.6	0.7	0.5	0.6	0.6
**Current hormone use (%)** ^b^	28.3	30.7	31.0	29.6	29.9	41.3	43.0	42.0	40.5	41.7
**Smoking status (%)**										
Current smoker	25.1	15.7	13.4	13.1	16.7	13.2	7.4	6.0	5.4	7.8
**Smoking, total cigarettes/d**	15.3 (9.9)	13.7 (9.7)	13.0 (9.8)	13.3 (9.6)	14.0 (9.8)	11.4 (8.2)	10.2 (7.6)	9.9 (7.9)	10.3 (8.4)	10.6 (8.0)
**Physical activity MET-hours/week**	14.0 (21.0)	16.2 (22.1)	16.9 (21.7)	16.7 (22.0)	16.0 (21.8)	14.7 (18.7)	18.2 (21.0)	20.5 (21.9)	22.5 (23.9)	19.1 (21.7)
**Alcohol, g/d**	5.9 (10.6)	5.5 (9.6)	5.2 (9.1)	4.6 (8.7)	5.3 (9.5)	5.9 (10.9)	6.1 (10.0)	6.8 (10.7)	7.7 (12.4)	6.6 (11.1)
**Diet quality, AHEI**	50.8 (10.9)	53.0 (11.0)	53.6 (11.0)	53.4 (11.1)	52.7 (11.0)	52.2 (10.9)	54.9 (11.5)	57.4 (12.0)	59.4 (12.8)	56.1 (12.2)
**Multivitamin use (%)**	35.7	39.3	39.3	38.4	37.7	64.2	69.3	71.1	72.3	69.1
**Depressed (%)**	7.7	7.9	7.0	7.4	7.5	11.4	10.5	10.3	10.6	10.7
**Total energy, kcal/d**	1489.1 (431.6)	1754.0 (458.7)	1847.3 (506.4)	1916.6 (528.3)	1755.6 (508.9)	1389.7 (417.5)	1647.0 (451.9)	1817.5 (506.1)	1974.5 (553.3)	1717.7 (533.6)
**Cereal fibre, g/d**	4.6 (3.3)	5.7 (3.7)	6.0 (3.8)	6.2 (3.7)	5.6 (3.7)	5.6 (3.3)	6.8 (3.6)	7.5 (4.1)	8.0 (4.3)	7.0 (4.0)
**Total saturated fat, g/d**	18.9 (7.8)	20.8 (8.0)	21.4 (8.2)	22.1 (8.5)	20.8 (8.2)	16.5 (7.6)	17.9 (8.0)	18.9 (8.5)	19.9 (8.6)	18.4 (8.3)
**Total polyunsaturated fat, g/d**	10.0 (4.3)	11.6 (4.6)	12.0 (4.7)	12.5 (5.0)	11.6 (4.7)	9.4 (4.4)	10.8 (4.8)	12.0 (5.6)	13.1 (6.2)	11.4 (5.5)
**Total folate, mcg/d**	364.6 (219.6)	453.9 (234.7)	480.6 (236.5)	507.9 (240.6)	453.1 (239.4)	636.4 (302.2)	753.1 (311.2)	827.9 (330.2)	891.3 (353.5)	781.7 (339.0)
**Total trans fat, g/d**	2.7 (1.4)	2.9 (1.5)	3.0 (1.5)	3.1 (1.5)	2.9 (1.5)	2.0 (1.0)	2.1 (1.1)	2.1 (1.2)	2.2 (1.2)	2.1 (1.1)
**Sugar soft drinks, serves/d**	0.2 (0.6)	0.2 (0.5)	0.2 (0.5)	0.3 (0.5)	0.2 (0.5)	0.2 (0.5)	0.2 (0.5)	0.3 (0.6)	0.3 (0.7)	0.3 (0.6)
**Nuts, serves/d**	0.2 (0.3)	0.2 (0.3)	0.2 (0.3)	0.2 (0.4)	0.2 (0.3)	0.2 (0.3)	0.3 (0.4)	0.4 (0.5)	0.5 (0.6)	0.4 (0.5)
**Animal meat, serves/d**	1.1 (0.8)	1.1 (0.8)	1.2 (0.8)	1.2 (0.8)	1.2 (0.8)	1.0 (0.7)	1.0 (0.7)	1.0 (0.7)	1.0 (0.7)	1.0 (0.7)

Values are expressed as mean (standard deviation [SD]) or range) for continuous variables and percentages for categorical variables.

All values are standardized to the age distribution of the study population.

Values of polytomous variables may not sum to 100 % due to rounding.

a Value is not age adjusted.

b Among postmenopausal women only.

**Table 2:** Multivariable generalized estimating equations with a Poisson distribution evaluating the association between the flavodiet score and flavonoid-rich food intake with likelihood of reporting sustained high levels of happiness (n = 44,659) and optimism (n = 36,723).

Sustained High Happiness Between 1992 and 2000						
Quartiles of intake						
	Q1	Q2	Q3	Q4	P-trend	P-corrected
**Flavodiet score**						
Cases/total	7362/10,867	8472/11,804	8089/10,879	8055/11,109		
Intake, servings/d^a^	0.28 (0.0–0.56)	0.86 (0.57–1.14)	1.5 (1.14–2.0)	3.0 (2.0–13.57)		
RR (95 % CI)						
Model 1	1.00^b^	1.06 (1.04–1.08)	1.08 (1.07–1.10)	1.06 (1.05–1.08)	<0.0001	
Model 2	1.00^b^	1.04 (1.02–1.06)	1.05 (1.04–1.07)	1.03 (1.02–1.05)	0.006	0.01
**Strawberry**						
Cases/total	7126/10,493	13,534/18,843	8578/11,705	2740/3618		
Intake, servings/d^a^	0.0 (0.0–0.0)	0.07 (0.07–0.07)	0.14 (0.14–0.14)	0.43 (0.43–6.0)		
RR (95 % CI)						
Model 1	1.00^b^	1.06 (1.04–1.07)	1.07 (1.06–1.09)	1.11 (1.08–1.13)	<0.0001	
Model 2	1.00^b^	1.05 (1.03–1.07)	1.06 (1.04–1.08)	1.08 (1.05–1.10)	<0.0001	0.0004
**Blueberry**						
Cases/total	19,413/27,526	8440/11,597	3356/4516	769/1020		
Intake, servings/d^a^	0.0 (0.0–0.0)	0.07 (0.07–0.07)	0.14 (0.14–0.14)	0.43 (0.43–6.0)		
RR (95 % CI)						
Model 1	1.00^b^	1.03 (1.02–1.05)	1.05 (1.03–1.07)	1.06 (1.03–1.10)	<0.0001	
Model 2	1.00^b^	1.02 (1.00–1.03)	1.02 (1.01–1.04)	1.03 (0.99–1.07)	0.05	0.07
**Tea**						
Cases/total	11,356/15,737	4356/6175	7238/10,116	9028/12,631		
Intake, servings/d^a^	0.0 (0.0–0.0)	0.07 (0.07–0.07)	0.43 (0.14–0.79)	2.5 (1.0–6.0)		
RR (95 % CI)						
Model 1	1.00^b^	0.98 (0.96–1.00)	0.99 (0.98–1.01)	0.99 (0.98–1.01)	0.07	
Model 2	1.00^b^	0.98 (0.96–1.00)	0.99 (0.97–1.01)	0.99 (0.98–1.01)	0.16	0.18
**Red wine**						
Cases/total	24,291/34,146	6220/8532	989/1340	478/641		
Intake, servings/d^a^	0.0 (0.0–0.0)	0.07 (0.07–0.14)	0.43 (0.43–0.43)	1.0 (0.79–6.0)		
RR (95 % CI)						
Model 1	1.00^b^	1.03 (1.01–1.04)	1.04 (1.00–1.07)	1.05 (1.00–1.10)	0.06	
Model 2	1.00^b^	1.02 (1.01–1.04)	1.02 (0.99–1.06)	1.04 (0.99–1.09)	0.22	0.23
**Apple**						
Cases/total	10,776/15,668	6802/9459	9180/12,522	5220/7010		
Intake, servings/d^a^	0.07 (0.0–0.07)	0.14 (0.14–0.14)	0.43 (0.43–0.43)	1.0 (0.79–6.0)		
RR (95 % CI)						
Model 1	1.00^b^	1.04 (1.03–1.06)	1.06 (1.04–1.08)	1.07 (1.05–1.09)	<0.0001	
Model 2	1.00^b^	1.03 (1.01–1.05)	1.03 (1.02–1.05)	1.03 (1.01–1.05)	0.05	0.07
**Orange**						
Cases/total	7947/11,639	9096/12,801	6386/8741	8549/11,478		
Intake, servings/d^a^	0.0 (0.0–0.0)	0.07 (0.07–0.07)	0.14 (0.14–0.14)	0.43 (0.43–6.0)		
RR (95 % CI)						
Model 1	1.00^b^	1.04 (1.03–1.06)	1.07 (1.05–1.09)	1.08 (1.07–1.10)	<0.0001	
Model 2	1.00^b^	1.04 (1.02–1.05)	1.06 (1.04–1.08)	1.06 (1.04–1.08)	<0.0001	0.0004
**Grapefruit**						
Cases/total	13,327/19,460	9445/12,978	5206/6971	4000/5250		
Intake, servings/d^a^	0.0 (0.0–0.0)	0.07 (0.07–0.07)	0.14 (0.14–0.14)	0.43 (0.43–6.0)		
RR (95 % CI)						
Model 1	1.00^b^	1.06 (1.05–1.08)	1.08 (1.06–1.10)	1.09 (1.07–1.11)	<0.0001	
Model 2	1.00^b^	1.05 (1.03–1.06)	1.06 (1.04–1.08)	1.06 (1.04–1.08)	0.0002	0.0005
Sustained high optimism between 2004 and 2012						
Quartiles of intake						
	Q1	Q2	Q3	Q4	P-trend	P-corrected
**Flavodiet score**						
Cases/total	2479/8915	2822/9468	3069/9162	3149/9178		
Intake, servings/d^a^	0.28 (0.0–0.57)	0.86 (0.57–1.16)	1.51 (1.16–2.08)	3.08 (2.09–14.5)		
RR (95 % CI)						
Model 1	1.00^b^	1.07 (1.02–1.12)	1.15 (1.10–1.20)	1.16 (1.11–1.21)	<0.0001	
Model 2	1.00^b^	1.03 (0.98–1.08)	1.07 (1.02–1.12)	1.06 (1.01–1.11)	0.03	0.05
**Strawberry**						
Cases/total	2442/9152	4419/14,115	2985/8944	1673/4512		
Intake, servings/d^a^	0.0 (0.0–0.0)	0.07 (0.07–0.07)	0.14 (0.14–0.14)	0.43 (0.43–6.0)		
RR (95 % CI)						
Model 1	1.00^b^	1.10 (1.05–1.15)	1.17 (1.12–1.22)	1.24 (1.18–1.31)	<0.0001	
Model 2	1.00^b^	1.08 (1.04–1.13)	1.13 (1.08–1.18)	1.16 (1.10–1.22)	<0.0001	0.0004
**Blueberry**						
Cases/total	5094/18,050	3426/10,342	1858/5352	1141/2979		
Intake, servings/d^a^	0.0 (0.0–0.0)	0.07 (0.07–0.07)	0.14 (0.14–0.14)	0.43 (0.43–6.0)		
RR (95 % CI)						
Model 1	1.00^b^	1.10 (1.06–1.14)	1.17 (1.12–1.22)	1.27 (1.20–1.33)	<0.0001	
Model 2	1.00^b^	1.06 (1.02–1.10)	1.11 (1.06–1.16)	1.14 (1.08–1.21)	<0.0001	0.0004
**Tea**						
Cases/total	2967/9937	2549/7997	2654/8416	3349/10,373		
Intake, servings/d^a^	0.0 (0.0–0.0)	0.07 (0.07–0.14)	0.43 (0.21–0.93)	2.0 (1.0–12.0)		
RR (95 % CI)						
Model 1	1.00^b^	1.04 (1.00–1.09)	1.04 (0.99–1.08)	1.03 (0.99–1.08)	0.88	
Model 2	1.00^b^	1.03 (0.98–1.07)	1.01 (0.97–1.06)	1.00 (0.96–1.04)	0.14	0.17
**Red wine**						
Cases/total	7198/23,976	2667/8019	1008/2871	646/1857		
Intake, servings/d^a^	0.0 (0.0–0.0)	0.07 (0.07–0.14)	0.43 (0.21–0.93)	1.0 (0.79–6.0)		
RR (95 % CI)						
Model 1	1.00^b^	1.02 (0.99–1.06)	1.07 (1.02–1.13)	1.08 (1.01–1.16)	0.02	
Model 2	1.00^b^	1.00 (0.96–1.03)	1.01 (0.95–1.06)	1.00 (0.93–1.08)	0.93	0.93
**Apple**						
Cases/total	1701/6341	2835/9586	2446/7666	4537/13,130		
Intake, servings/d^a^	0.0 (0.0–0.0)	0.07 (0.07–0.07)	0.14 (0.14–0.14)	0.43 (0.43–6.0)		
RR (95 % CI)						
Model 1	1.00^b^	1.06 (1.01–1.11)	1.14 (1.08–1.20)	1.23 (1.18–1.29)	<0.0001	
Model 2	1.00^b^	1.04 (0.99–1.10)	1.10 (1.05–1.16)	1.15 (1.09–1.21)	0.0002	0.0005
**Orange**						
Cases/total	3593/12,572	3247/10,357	2164/6423	2515/7371		
Intake, servings/d^a^	0.0 (0.0–0.0)	0.07 (0.07–0.07)	0.14 (0.14–0.14)	0.43 (0.43–6.0)		
RR (95 % CI)						
Model 1	1.00^b^	1.05 (1.01–1.09)	1.14 (1.09–1.19)	1.16 (1.11–1.21)	<0.0001	
Model 2	1.00^b^	1.04 (1.00–1.08)	1.11 (1.06–1.16)	1.10 (1.05–1.46)	0.05	0.07
**Grapefruit**						
Cases/total	6644/22,618	2327/6658	1168/3497	1380/3950		
Intake, servings/d^a^	0.0 (0.0–0.0)	0.05 (0.05–0.05)	0.09 (0.09–0.09)	0.29 (0.29–4.0)		
Model 1	1.00^b^	1.12 (1.08–1.16)	1.10 (1.04–1.15)	1.15 (1.10–1.20)	<0.0001	
Model 2	1.00^b^	1.11 (1.06–1.15)	1.07 (1.01–1.12)	1.10 (1.05–1.15)	0.0003	0.0007

Model 1 adjusted for age (calendar year), ethnicity, education and marital status.

Model 2: Model 1 plus smoking status, multivitamin use, physical activity, BMI, menopausal status, postmenopausal hormone use, and intakes of alcohol, total energy, meat, nuts, saturated fat, polyunsaturated fat, trans fat, cereal fibre, and soft drinks.

P-trend is for linear trend.

P-corrected is the P-trend corrected for multiple testing (Benjamin-Hochberg).

Abbreviations: Q, quartile; RR, risk ratio; CI, confidence interval; BMI, Body Mass Index.

a Median intakes (range).

b Reference categories.

**Table 3:** Multivariable generalized estimating equations with a Poisson distribution evaluating the association between flavonoid intake with likelihood of reporting sustained high levels of happiness (n = 44,659) and optimism (n = 36,723).

Sustained High Happiness Between 1992 and 2000						
Quartiles of intake						
	Q1	Q2	Q3	Q4	P-trend	P-corrected
**Total flavonoids**						
**Cases/total**	7664/11,160	8109/11,167	8167/11,168	8038/11,164		
Intake, mg/d^a^	107.4 (3.2–155.9)	203.4 (156.0–259.9)	345.2 (260.0–452.0)	828.0 (452.1–3017.4)		
RR (95 % CI)						
Model 1	1.00^b^	1.05 (1.03–1.07)	1.06 (1.04–1.08)	1.04 (1.03–1.06)	0.006	
Model 2	1.00^b^	1.03 (1.01–1.05)	1.04 (1.02–1.06)	1.03 (1.01–1.05)	0.40	0.51
**Flavonols**						
Cases/total	7669/11,157	8055/11,171	8222/11,167	8032/11,164		
Intake, mg/d^a^	7.4 (0.9–10.1)	12.6 (10.1–15.3)	19.0 (15.4–25.1)	34.4 (25.1–211.8)		
RR (95 % CI)						
Model 1	1.00^b^	1.05 (1.03–1.07)	1.06 (1.05–1.08)	1.04 (1.02–1.06)	0.001	
Model 2	1.00^b^	1.03 (1.02–1.05)	1.05 (1.03–1.06)	1.02 (1.00–1.04)	0.30	0.42
**Flavones**						
**Cases/total**	7431/11,101	7997/11,281	8072/11,057	8478/11,220		
Intake, mg/d^a^	0.6 (0.0–0.9)	1.2 (0.9–1.6)	2.1 (1.7–2.6)	3.1 (2.6–62.5)		
RR (95 % CI)						
Model 1	1.00^b^	1.06 (1.04–1.08)	1.08 (1.06–1.10)	1.11 (1.10–1.13)	<0.0001	
Model 2	1.00^b^	1.05 (1.03–1.07)	1.07 (1.05–1.09)	1.10 (1.08–1.12)	<0.0001	0.0002
**Flavanones**						
Cases/total	7487/11,169	7959/11,158	8123/11,166	8409/11,166		
Intake, mg/d^a^	6.6 (0.0–12.6)	24.1 (12.6–34.7)	52.0 (34.7–60.7)	75.7 (60.7–672.9)		
RR (95 % CI)						
Model 1	1.00^b^	1.06 (1.04–1.08)	1.08 (1.06–1.10)	1.11 (1.09–1.13)	<0.0001	
Model 2	1.00^b^	1.05 (1.03–1.07)	1.06 (1.05–1.08)	1.09 (1.07–1.11)	<0.0001	0.0002
**Flavan-3-ol monomers**						
Cases/total	7788/11,132	8137/11,201	8045/11,158	8008/11,168		
Intake, mg/d^a^	8.1 (0.1–12.6)	17.3 (12.7–24.7)	41.9 (24.8–74.0)	171.0 (74.1–478.2)		
RR (95 % CI)						
Model 1	1.00^b^	1.03 (1.02–1.05)	1.03 (1.01–1.05)	1.02 (1.00–1.04)	0.42	
Model 2	1.00^b^	1.01 (1.00–1.03)	1.01 (0.99–1.03)	1.01 (0.99–1.02)	0.97	0.97
**Anthocyanins**						
**Cases/total**	7573/11,150	7981/11,184	8096/11,148	8328/11,177		
Intake, mg/d^a^	1.8 (0.0–3.1)	4.4 (3.1–6.1)	12.6 (6.1–15.0)	21.9 (15.0–872.1)		
RR (95 % CI)						
Model 1	1.00^b^	1.04 (1.03–1.06)	1.06 (1.05–1.08)	1.09 (1.07–1.11)	<0.0001	
Model 2	1.00^b^	1.03 (1.02–1.05)	1.04 (1.03–1.06)	1.06 (1.04–1.08)	<0.0001	0.0002
**Flavan-3-ol polymers**						
**Cases/total**	7762/11,164	8048/11,166	8147/11,164	8021/11,165		
Intake, mg/d^a^	44.5 (2.3–71.8)	101.4 (71.8–138.0)	192.0 (138.0–261.9)	526.7 (261.9–1664.0)		
RR (95 % CI)						
Model 1	1.00^b^	1.03 (1.01–1.05)	1.04 (1.03–1.06)	1.03 (1.01–1.05)	0.05	
Model 2	1.00^b^	1.01 (1.00–1.03)	1.02 (1.01–1.04)	1.01 (0.99–1.03)	0.75	0.81
Sustained high optimism between 2004 and 2012						
Quartiles of intake						
	Q1	Q2	Q3	Q4	P-trend	P-corrected
**Total flavonoids**						
**Cases/total**	2574/9177	2825/9185	3047/9183	3073/9178		
Intake, mg/d^a^	124.9 (3.5–173.8)	221.5 (173.9–274.9)	339.0 (275.0–429.1)	596.9 (429.2–3141.7)		
RR (95 % CI)						
Model 1	1.00^b^	1.07 (1.02–1.12)	1.16 (1.11–1.21)	1.15 (1.10–1.20)	<0.0001	
Model 2	1.00^b^	1.05 (1.00–1.10)	1.12 (1.06–1.17)	1.09 (1.04–1.15)	0.004	0.007
**Flavonols**						
Cases/total	2497/9173	2783/9190	3072/9183	3167/9177		
Intake, mg/d^a^	7.8 (0.3–10.6)	13.1 (10.6–15.8)	19.1 (15.8–23.8)	32.5 (23.8–238.6)		
RR (95 % CI)						
Model 1	1.00^b^>	1.09 (1.04–1.14)	1.17 (1.12–1.23)	1.20 (1.15–1.26)	<0.0001	
Model 2	1.00^b^	1.07 (1.02–1.12)	1.13 (1.08–1.19)	1.15 (1.09–1.21)	<0.0001	0.0002
**Flavones**						
Cases/total	2573/9168	2831/9220	2957/9182	3158/9153		
Intake, mg/d^a^	0.8 (0.0–1.4)	1.9 (1.4–2.4)	2.9 (2.4–3.5)	4.6 (3.5–56.0)		
RR (95 % CI)						
Model 1	1.00^b^	1.08 (1.03–1.13)	1.15 (1.10–1.20)	1.22 (1.16–1.27)	<0.0001	
Model 2	1.00^b^	1.07 (1.02–1.12)	1.13 (1.08–1.18)	1.18 (1.12–1.24)	<0.0001	0.0002
**Flavanones**						
Cases/total	2735/9179	2905/9180	2831/9180	3048/9184		
Intake, mg/d^a^	5.3 (0.0–11.8)	23.8 (11.8–33.9)	52.4 (33.9–60.7)	73.2 (60.7–662.6)		
RR (95 % CI)						
Model 1	1.00^b^	1.06 (1.01–1.11)	1.07 (1.02–1.12)	1.15 (1.10–1.20)	<0.0001	
Model 2	1.00^b^	1.06 (1.01–1.11)	1.06 (1.01–1.11)	1.13 (1.08–1.18)	<0.0001	0.0002
**Flavan-3-ol monomers**						
Cases/total	2553/9185	2930/9179	3093/9179	2943/9180		
Intake, mg/d^a^	9.0 (0.0–13.3)	17.7 (13.3–23.0)	32.0 (23.0–48.2)	82.1 (48.2–462.7)		
RR (95 % CI)						
Model 1	1.00^b^	1.11 (1.06–1.16)	1.17 (1.12–1.23)	1.11 (1.06–1.16)	0.01	
Model 2	1.00^b^	1.08 (1.03–1.13)	1.11 (1.06–1.17)	1.06 (1.01–1.11)	0.69	0.81
**Anthocyanins**						
Cases/total	2396/9179	2716/9182	3041/9186	3366/9176		
Intake, mg/d^a^	3.4 (0.0–6.0)	10.5 (6.0–15.1)	19.4 (15.1–24.9)	35.5 (24.9–940.9)		
RR (95 % CI)						
Model 1	1.00^b^	1.09 (1.04–1.14)	1.20 (1.14–1.25)	1.29 (1.23–1.34)	<0.0001	
Model 2	1.00^b^	1.05 (1.00–1.11)	1.14 (1.08–1.19)	1.16 (1.11–1.23)	<0.0001	0.0002
**Flavan-3-ol polymers**						
Cases/total	2622/9179	2839/9182	3051/9181	3007/9181		
Intake, mg/d^a^	55.2 (1.7–84.0)	112.7 (84.0–145.0)	185.9 (145.0–242.2)	349.1 (242.3–1765.0)		
RR (95 % CI)						
Model 1	1.00^b^	1.06 (1.01–1.10)	1.14 (1.09–1.19)	1.10 (1.06–1.15)	<0.0001	
Model 2	1.00^b^	1.03 (0.98–1.08)	1.09 (1.04–1.14)	1.04 (0.99–1.09)	0.29	0.42

Model 1 adjusted for age (calendar year), ethnicity, education and marital status.

Model 2: Model 1 plus smoking status, multivitamin use, physical activity, BMI, menopausal status, postmenopausal hormone use, and intakes of alcohol, total energy, meat, nuts, saturated fat, polyunsaturated fat, trans fat, cereal fibre, and soft drinks.

P-trend is for linear trend.

P-corrected is the P-trend corrected for multiple testing (Benjamin-Hochberg).

Abbreviations: Q, quartile; RR, risk ratio; CI, confidence interval; BMI, Body Mass Index.

a Median intakes (range).

b Reference categories.

**Table 4:** Multivariable generalized estimating equations with a Poisson distribution evaluating the association between baseline levels of happiness (n = 43,564) and optimism (n = 30,823) with likelihood of reporting sustained flavonoid intake.

Baseline Happiness in 1992				
	Lower	Moderate	Higher	P-trend
**Sustained flavodiet score (1994–2010)**				
Cases/total	3547/6250	4842/8118	17,937/29,196	
RR (95 % CI)				
Model 1	1.00^a^	1.06 (1.03–1.09)	1.11 (1.08–1.13)	<0.0001
Model 2	1.00^a^	1.04 (1.01–1.07)	1.06 (1.03–1.08)	<0.0001
**Sustained flavonoid intake (1994–2010)**				
Cases/total	3627/6250	4941/8118	18,068/29,196	
RR (95 % CI)				
Model 1	1.00^a^	1.06 (1.03–1.09)	1.09 (1.06–1.11)	<0.0001
Model 2	1.00^a^	1.04 (1.01–1.07)	1.05 (1.03–1.07)	<0.0001
Baseline optimism in 2004				
	Lower	Moderate	Higher	P-trend
**Sustained flavodiet score (2006–2010)**				
Cases/total	3256/9649	3680/9487	4763/11,687	
RR (95 % CI)				
Model 1	1.00^a^	1.13 (1.09–1.17)	1.16 (1.12–1.21)	<0.0001
Model 2	1.00^a^	1.09 (1.05–1.13)	1.09 (1.05–1.13)	<0.0001
**Sustained flavonoid intake (2006–2010)**				
Cases/total	3374/9649	3682/9487	4666/11,687	
RR (95 % CI)				
Model 1	1.00^a^	1.09 (1.05–1.13)	1.11 (1.07–1.14)	<0.0001
Model 2	1.00^a^	1.06 (1.02–1.10)	1.05 (1.01–1.08)	0.007

Model 1 adjusted for age (calendar year), ethnicity, education and marital status.

Model 2: Model 1 plus smoking status, multivitamin use, physical activity, BMI, menopausal status, postmenopausal hormone use, and intakes of alcohol, total energy, meat, nuts, saturated fat, polyunsaturated fat, trans fat, cereal fibre, and soft drinks.

P-trend is for linear trend.

Abbreviations: Q, quartile; RR, risk ratio; CI, confidence interval; BMI, Body Mass Index.

a Reference categories.

## Data Availability

Data described in the article, code book, and analytic code will be made available upon request pending approval by the Channing Division of Network Medicine at Brigham and Women's Hospital and Harvard Medical School. Further information including the procedures to obtain and access data from the Nurses' Health Study is described at https://www.nurseshealthstudy.org/researchers (contact: nhsaccess@channing.harvard.edu).

## Appendix A. Supplementary data

Supplementary data to this article can be found online at https://doi.org/10.1016/j.clnu.2026.106579.
