# Supplementary material for: Dietary flavonoid intake and psychological well-being – A bidirectional relationship
Source: Clin Nutr. Author manuscript; Available in PMC 2026 Jul 10. (PMC13352143; doi:10.1016/j.clnu.2026.106579)
Supplement: 1 [file NIHMS2189757-supplement-1.docx]

**Dietary flavonoid intake and psychological well-being – a bidirectional relationship**

Supplementary Material

*Thompson et al.*

**Table S1.** Summary of key measures, definitions, and use in analysis

| **Measure / Endpoint** | **Assessment Years** | **Scale or Measure** | **Definition / Categorization** | **Use in Analysis** |
| --- | --- | --- | --- | --- |
| Happiness | 1992, 1996, 2000 | 6-point Likert scale (1=all the time to 6 = none of the time) | Tertiles: Higher (1–2), Moderate (3), Lower (4–6) | Binary variable for sustained high happiness (≥2 of 3 timepoints in highest tertile); also used as 3-level ordinal variable |
| Stricter Sustained Happiness (Sensitivity Analysis) | 1992, 1996, 2000 | As above | High happiness at all 3 timepoints | Sensitivity analysis of primary outcome |
| Optimism | 2004, 2008, 2012 | Life Orientation Test-Revised (score 0-30) | Tertiles: Higher (28–30), Moderate (24–27), Lower (0–23) | Binary variable for sustained high optimism (≥2 of 3 timepoints in highest tertile); also used as 3-level ordinal variable |
| Stricter Sustained Optimism (Sensitivity Analysis) | 2004, 2008, 2012 | As above | High optimism at all 3 timepoints | Sensitivity analysis of primary outcome |
| Flavodiet Score | Baseline (1990), Follow-up (1994-2010) | Composite score from 7 flavonoid-rich food items | Combined intake of apples, blueberries, oranges/orange juice, strawberries, grapefruit, tea, red wine | Exposure variable (baseline and sustained intake) |
| Total Flavonoid Intake | Baseline (1990), Follow-up (1994-2010) | Mg/day, derived from FFQs linked to USDA flavonoid database | Summed across all flavonoid subclasses | Exposure variable (baseline and sustained intake) |
| Sustained Flavodiet / Flavonoid Intake | 1994-2010 (happiness), 2006-2010 (optimism) | Quartiles | Remaining in top three quartiles (Q3–Q4) across all assessments | Outcome in bidirectional analyses |

| **Table S2.** Multivariable generalized estimating equations with a Poisson distribution evaluating the association between the flavodiet score and total flavonoid intake with likelihood of reporting sustained high levels of happiness (n=44,659) and optimism (n=36,723), stratified by BMI groups | | | | | | | |  |  |  |
| --- | --- | --- | --- | --- | --- | --- | --- | --- | --- | --- |
|  | **Sustained High Happiness Between 1992 and 2000** | | | | | |  |  |  |  |
|  | **Quartiles of Intake** | | | | | |  |  |  |  |
|  | | **Q1** | **Q2** | **Q3** | **Q4** | **P-trend** | **P-interaction** |  |  |  |
| **Flavodiet Score** | |  |  |  |  |  |  |  |  |  |
| **BMI <25.0 kg/m^2^** | |  |  |  |  |  | 0.86 |  |  |  |
| Cases/total | | 4,199/6,055 | 4,737/6,503 | 4,590/6,061 | 4,593/6,248 |  |  |  |  |  |
| Intake, servings/d^a^ | | 0.3 (0.0-0.6) | 0.9 (0.6-1.1) | 1.5 (1.1-2.0) | 3.0 (2.0-13.6) |  |  |  |  |  |
| RR (95% CI)^b^ | | 1.00^c^ | 1.03 (1.00-1.05) | 1.05 (1.02-1.07) | 1.02 (1.00-1.05) | 0.17 |  |  |  |  |
| **BMI ≥ 25.0 kg/m^2^** | |  |  |  |  |  |  |  |  |  |
| Cases/total | | 3,163/4,812 | 3,735/5,301 | 3,499/4,818 | 3,462/4,861 |  |  |  |  |  |
| Intake, servings/d^a^ | | 0.3 (0.0-0.6) | 0.9 (0.6-1.1) | 1.5 (1.1-2.0) | 3.0 (2.0-12.1) |  |  |  |  |  |
| RR (95% CI)^b^ | | 1.00^c^ | 1.05 (1.02-1.08) | 1.06 (1.03-1.09) | 1.05 (1.02-1.08) | 0.01 |  |  |  |  |
| **Total Flavonoids** | |  |  |  |  |  |  |  |  |  |
| **BMI <25.0 kg/m^2^** | |  |  |  |  |  | 0.47 |  |  |  |
| Cases/total | | 4,290/6,116 | 4,624/6,261 | 4,551/6,158 | 4,654/6,332 |  |  |  |  |  |
| Intake, servings/d^a^ | | 107.2 (3.2-155.9) | 203.4 (156.0-259.9) | 345.6 (260.0-452.0) | 825.8 (452.1-3017.4) |  |  |  |  |  |
| RR (95% CI)^b^ | | 1.00^c^ | 1.03 (1.00-1.05) | 1.03 (1.00-1.05) | 1.02 (1.00-1.05) | 0.47 |  |  |  |  |
| **BMI ≥ 25.0 kg/m^2^** | |  |  |  |  |  |  |  |  |  |
| Cases/total | | 3,374/5,044 | 3,485/4,906 | 3,616/5,010 | 3,384/4,832 |  |  |  |  |  |
| Intake, servings/d^a^ | | 107.7 (4.9-155.9) | 203.4 (156.0-259.9) | 344.6 (260.0-452.0) | 831.9 (452.1-2375.2) |  |  |  |  |  |
| RR (95% CI)^b^ | | 1.00^c^ | 1.04 (1.01-1.07) | 1.06 (1.03-1.09) | 1.03 (1.00-1.06) | 0.66 |  |  |  |  |
|  | **Sustained High Optimism Between 2004 and 2012** | | | | | |  |  |  |  |
|  | **Quartiles of Intake** | | | | | |  |  |  | **Quartiles of Intake** |
|  | | **Q1** | **Q2** | **Q3** | **Q4** | **P-trend** | **P-interaction** |  |  |  |
| **Flavodiet Score** | |  |  |  |  |  |  |  |  |  |
| **BMI <25.0 kg/m^2^** | |  |  |  |  |  | 0.26 |  |  |  |
| Cases/total | | 1,083/3,799 | 1,313/4,195 | 1,458/4,259 | 1,564/4,482 |  |  |  |  |  |
| Intake, servings/d^a^ | | 0.3 (0.0-0.6) | 0.9 (0.6-1.2) | 1.5 (1.2-2.1) | 3.1 (2.1-14.5) |  |  |  |  |  |
| RR (95% CI)^b^ | | 1.00^c^ | 1.06 (0.98-1.13) | 1.06 (0.98-1.13) | 1.04 (0.97-1.12) | 0.49 |  |  |  |  |
| **BMI ≥ 25.0 kg/m^2^** | |  |  |  |  |  |  |  |  |  |
| Cases/total | | 1,396/5,116 | 1,509/5,273 | 1,611/4,903 | 1,585/4,696 |  |  |  |  |  |
| Intake, servings/d^a^ | | 0.3 (0.0-0.6) | 0.9 (0.6-1.2) | 1.5 (1.2-2.1) | 3.1 (2.1-13.0) |  |  |  |  |  |
| RR (95% CI)^b^ | | 1.00^c^ | 1.00 (0.94-1.07) | 1.08 (1.01-1.15) | 1.07 (1.00-1.14) | 0.02 |  |  |  |  |
| **Total Flavonoids** | |  |  |  |  |  |  |  |  |  |
| **BMI <25.0 kg/m^2^** | |  |  |  |  |  | 0.12 |  |  |  |
| Cases/total | | 1,146/3,885 | 1,298/4,072 | 1,451/4,256 | 1,523/4,522 |  |  |  |  |  |
| Intake, servings/d^a^ | | 125.8 (3.5-173.8) | 222.9 (173.9-274.9) | 339.3 (275.0-429.1) | 597.9 (429.2-3141.7) |  |  |  |  |  |
| RR (95% CI)^b^ | | 1.00^c^ | 1.03 (0.96-1.11) | 1.09 (1.02-1.17) | 1.03 (0.96-1.11) | 0.81 |  |  |  |  |
| **BMI ≥ 25.0 kg/m^2^** | |  |  |  |  |  |  |  |  |  |
| Cases/total | | 1,428/5,292 | 1,527/5,113 | 1,596/4,927 | 1,550/4,656 |  |  |  |  |  |
| Intake, servings/d^a^ | | 124.4 (6.2-173.8) | 220.1 (173.9-274.9) | 338.8 (275.0-429.1) | 595.8 (429.2-2306.6) |  |  |  |  |  |
| RR (95% CI)^b^ | | 1.00^c^ | 1.06 (0.99-1.13) | 1.13 (1.06-1.21) | 1.14 (1.06-1.22) | 0.0002 |  |  |  |  |
| ^a^Median intakes (range)  ^b^Risk Ratios with 95% Confidence Intervals (CI), adjusted for age (calendar year), ethnicity, education and marital status, smoking status, multivitamin use, physical activity, menopausal status, postmenopausal hormone use, and intakes of alcohol, total energy, meat, nuts, saturated fat, polyunsaturated fat, trans fat, cereal fibre, and soft drinks.  ^c^Reference categories.  P-trend is for linear trend  P-interaction values were obtained using likelihood ratio tests, comparing models with and without an interaction term between BMI category (<25 vs. ≥25) and flavonoid intake.  *Abbreviations: Q, quartile; RR, risk ratio; CI, confidence interval; BMI, Body Mass Index.* | | | | | | | |  |  |  |

| **Table S3.** Sensitivity analysis showing generalized estimating equations with a Poisson distribution evaluating the association between the flavodiet score and total flavonoid intake with likelihood of reporting sustained high levels of happiness (n=41,306) and optimism (n=32,795) excluding participants with depression at baseline | | | | | | |
| --- | --- | --- | --- | --- | --- | --- |
|  | **Sustained High Happiness Between 1992 and 2000** | | | | | |
|  | **Quartiles of Intake** | | | | | |
|  | | **Q1** | **Q2** | **Q3** | **Q4** | **P-trend** |
| **Flavodiet Score** | |  |  |  |  |  |
| Cases/total | | 7,039/10,008 | 8,081/10,910 | 7,712/10,102 | 7,699/10,286 |  |
| Intake, servings/d^a^ | | 0.3 (0.0-0.6) | 0.9 (0.6-1.1) | 1.5 (1.1-2.0) | 3.0 (2.0-13.6) |  |
| RR (95% CI)^b^ | | 1.00^c^ | 1.03 (1.02-1.05) | 1.04 (1.03-1.06) | 1.03 (1.01-1.05) | 0.01 |
| **Total Flavonoids** | |  |  |  |  |  |
| Cases/total | | 7,331/10,298 | 7,725/10,281 | 7,789/10,396 | 7,686/10,331 |  |
| Intake, mg/d^a^ | | 107.4 (3.2-155.9) | 203.4 (156.0-259.9) | 345.2 (260.0-452.0) | 828.0 (452.1-3017.4) |  |
| RR (95% CI)^b^ | | 1.00^c^ | 1.03 (1.01-1.05) | 1.03 (1.01-1.05) | 1.02 (1.00-1.04) | 0.51 |
|  | **Sustained High Optimism Between 2004 and 2012** | | | | | |
|  | **Quartiles of Intake** | | | | | |
|  | | **Q1** | **Q2** | **Q3** | **Q4** | **P-trend** |
| **Flavodiet Score** | |  |  |  |  |  |
| Cases/total | | 2,302/7,870 | 2,634/8,477 | 2,862/8,239 | 2,951/8,209 |  |
| Intake, servings/d^a^ | | 0.3 (0.0-0.6) | 0.9 (0.6-1.2) | 1.5 (1.2-2.1) | 3.1 (2.1-14.5) |  |
| RR (95% CI)^b^ | | 1.00^c^ | 1.02 (0.97-1.07) | 1.06 (1.01-1.11) | 1.05 (1.00-1.11) | 0.03 |
| **Total Flavonoids** | |  |  |  |  |  |
| Cases/total | | 2,411/8,148 | 2,627/8,223 | 2,832/8,225 | 2,879/8,199 |  |
| Intake, mg/d^a^ | | 124.9 (3.5-173.8) | 221.5 (173.9-274.9) | 339.0 (275.0-429.1) | 596.9 (429.2-3141.7) |  |
| RR (95% CI)^b^ | | 1.00^c^ | 1.03 (0.98-1.08) | 1.09 (1.04-1.15) | 1.08 (1.03-1.14) | 0.004 |
| ^a^Median intakes (range)  ^b^Risk Ratios with 95% Confidence Intervals (CI), adjusted for age (calendar year), ethnicity, education and marital status, smoking status, multivitamin use, physical activity, BMI, menopausal status, postmenopausal hormone use, and intakes of alcohol, total energy, meat, nuts, saturated fat, polyunsaturated fat, trans fat, cereal fibre, and soft drinks.  ^c^Reference categories.  P-trend is for linear trend  *Abbreviations: Q, quartile; RR, risk ratio; CI, confidence interval; BMI, Body Mass Index.* | | | | | | |

| **Table S4.** Sensitivity analysis showing generalized estimating equations with a Poisson distribution evaluating the association between the flavodiet score and total flavonoid intake with likelihood of reporting sustained high levels of happiness (n=44,659) and optimism (n=36,723), based on a stricter definition requiring high levels at all assessment time points | | | | | | | | | | |
| --- | --- | --- | --- | --- | --- | --- | --- | --- | --- | --- |
|  | **Sustained High Happiness Between 1992 and 2000** | | | | | | | | | |
|  | **Quartiles of Intake** | | | | | | | | | |
|  | | **Q1** | | **Q2** | | **Q3** | | **Q4** | | **P-trend** |
| **Flavodiet Score** |  | |  | |  | |  | |  | |
| Cases/total | | 4,668/10,867 | | 5,534/11,804 | | 5,319/10,879 | | 5,335/11,109 | |  |
| Intake, servings/d^a^ | | 0.3 (0.0-0.6) | | 0.9 (0.6-1.1) | | 1.5 (1.1-2.0) | | 3.0 (2.0-13.6) | |  |
| RR (95% CI)^b^ | | 1.00^c^ | | 1.05 (1.02-1.08) | | 1.07 (1.04-1.11) | | 1.06 (1.03-1.09) | | 0.003 |
| **Total Flavonoids** | |  | |  | |  | |  | |  |
| Cases/total | | 4,895/11,160 | | 5,266/11,167 | | 5,401/11,168 | | 5,294/11,164 | |  |
| Intake, mg/d^a^ | | 107.4 (3.2-155.9) | | 203.4 (156.0-259.9) | | 345.2 (260.0-452.0) | | 828.0 (452.1-3017.4) | |  |
| RR (95% CI)^b^ | | 1.00^c^ | | 1.04 (1.01-1.07) | | 1.06 (1.03-1.10) | | 1.05 (1.02-1.08) | | 0.10 |
|  | **Sustained High Optimism Between 2004 and 2012** | | | | | | | | | |
|  | **Quartiles of Intake** | | | | | | | | | |
|  | | **Q1** | | **Q2** | | **Q3** | | **Q4** | | **P-trend** |
| **Flavodiet Score** | |  | |  | |  | |  | |  |
| Cases/total | | 1,029/8,915 | | 1,246/9,468 | | 1,397/9,162 | | 1,396/9,178 | |  |
| Intake, servings/d^a^ | | 0.3 (0.0-0.6) | | 0.9 (0.6-1.2) | | 1.5 (1.2-2.1) | | 3.1 (2.1-14.5) | |  |
| RR (95% CI)^b^ | | 1.00^c^ | | 1.09 (1.01-1.18) | | 1.14 (1.05-1.23) | | 1.08 (0.99-1.17) | | 0.21 |
| **Total Flavonoids** | |  | |  | |  | |  | |  |
| Cases/total | | 1,084/9,177 | | 1,282/9,185 | | 1,365/9,183 | | 1,337/9,178 | |  |
| Intake, mg/d^a^ | | 124.9 (3.5-173.8) | | 221.5 (173.9-274.9) | | 339.0 (275.0-429.1) | | 596.9 (429.2-3141.7) | |  |
| RR (95% CI)^b^ | | 1.00^c^ | | 1.09 (1.01-1.18) | | 1.16 (1.07-1.25) | | 1.09 (1.00-1.19) | | 0.26 |
| ^a^Median intakes (range)  ^b^Risk Ratios with 95% Confidence Intervals (CI), adjusted for age (calendar year), ethnicity, education and marital status, smoking status, multivitamin use, physical activity, BMI, menopausal status, postmenopausal hormone use, and intakes of alcohol, total energy, meat, nuts, saturated fat, polyunsaturated fat, trans fat, cereal fibre, and soft drinks.  ^c^Reference categories.  P-trend is for linear trend  *Abbreviations: Q, quartile; RR, risk ratio; CI, confidence interval; BMI, Body Mass Index.* | | | | | | | | | | |
